# A 20-year analysis of gender trends in oncology authorship in the Cochrane database of systematic reviews

**DOI:** 10.3389/fonc.2024.1450475

**Published:** 2024-10-15

**Authors:** Morgan S. Levy, Thilani Samarakoon, Caleigh Smith, Irene Goo, Lunthita M. Duthely, Maria Van Zuilen, Marilyn Huang, Asha B. Pillai

**Affiliations:** ^1^ Sylvester Comprehensive Cancer Center, University of Miami, Miami, FL, United States; ^2^ Department of Medical Education, University of Miami Miller School of Medicine, Miami, FL, United States; ^3^ Department of Health Informatics, Louis Calder Memorial Library, University of Miami Miller School of Medicine, Miami, FL, United States; ^4^ Department of Gynecologic Oncology, University of Virginia School of Medicine, Charlottesville, VA, United States; ^5^ Department of Public Health Sciences, University of Miami Miller School of Medicine, Miami, FL, United States; ^6^ Department of Obstetrics, Gynecology & Reproductive Sciences, Miller School of Medicine, Miami, FL, United States; ^7^ Department of Obstetrics, Gynecology & Reproductive Sciences, Division of Gynecologic Oncology, Miller School of Medicine, Miami, FL, United States; ^8^ Department of Pediatrics, University of Miami Miller School of Medicine, Miami, FL, United States

**Keywords:** gender inequity, academic medicine, oncology, Cochrane database of systematic reviews, women in medicine, authorship

## Abstract

**Objectives:**

The objective of this study was to evaluate global longitudinal publication trends in oncology in the Cochrane Database of Systematic Reviews (CDSR) from 2001-2020.

**Design:**

Retrospective bibliometric analysis.

**Primary and secondary outcome measures:**

The primary outcome measures were the numbers and percentages of women as first, last, and corresponding author across all CDSR oncology publications. Additional outcomes included authorship differences between countries and percentages of women authors over time compared using the Cochran-Armitage trend test.

**Results:**

In total, 548 articles were analyzed. Women were first authors in 52.26% (n=277) and corresponding authors in 50.75% (n=272), respectively. Women represented only 39.4% (n=210) of last authors, significantly less frequent than male counterparts (p < 0.001). The percentage of women last and corresponding authors has increased significantly in the past 20 years (p < 0.05). Countries such as the Netherlands and Australia consistently showed equitable representation in first, corresponding, and last authorship, while other countries such as Italy and China had uniformly low rates of female authorship.

**Coclusions:**

Our results highlight patterns of gender inequity in oncology publication authorship in the CDSR from 2001-2020 at a global level. Notably, women were less likely to serve in the last author position which, independent of assigned corresponding authorship, is generally assumed in academic oncology to designate the leader of a published study. Substantive efforts to correct this disparity are needed to achieve gender parity in publicly perceived leadership in oncology publications.

## Introduction

Gender inequity among academic leaders has been recognized for decades on an international scale. Across academic medicine, women face various challenges to attaining visibility of their scholarship and advancing to leadership roles. Globally, these inequities occur at every level of the scholarship ladder, starting from high impact publications. From 2002 to 2019, women were less likely than men to be the designated lead authors in top medical journals such as the *New England Journal of Medicine* (15.8%), the *Lancet* (29.4%), and the *Journal of the American Medical Association* (35.4%) (1). In oncology, a 2020 bibliometric analysis of five heavily cited journals (*Annals of Surgical Oncology; Cancer; International Journal of Radiation Oncology, Biology, Physics; JAMA Oncology;* and *Journal of Clinical Oncology*) found that women represented only 28.4% and 20.7% of first and last authors, respectively (2). Another analysis of articles in the *Journal of Clinical Global Oncology* from 2015 to 2020 showed that only 37.8% of authors were women (3). Published manuscripts led by women are also less likely to be cited in high-impact journals (4).

Scholarly output is a key metric evaluated in promotion, tenure, and leadership considerations, so such authorship inequities impede career advancement and may directly contribute to the underrepresentation of women in academic leadership. As of 2020, women represented only 13.3% (n = 85) of cancer center directors in the United States (5). Women are underrepresented on editorial boards for major hematology and oncology journals. In a recent study of 793 editorial board members representing 60 journals, only 27.4% of editors were women (6). While women and men are equally represented in clinical medical practice, women are underrepresented in academic professorships and leadership roles across most medical and surgical disciplines (7). Women constitute only 17.9% of all corresponding authors of oncology phase III clinical trial reports (8), where recognition of their clinical and scholarly efforts is essential to their advancement as leaders in academic oncology (9).

Recognizing existing disparities in the attainment of scholarly output by women in oncology, innovative strategies to promote high-quality work with minimal barriers are critical. In 2023, only 39% of all NIH grant funding was awarded to women (10). Systematic reviews hold a key fiscal advantage for unfunded researchers because their research does not require a laboratory setup. One equity-driving platform that catalogs high-quality systematic research reviews by subject matter is the Cochrane Database of Systematic Reviews (CDSR) (11). CDSR adheres to open-access publishing (no publication or reader access fee), addressing the most prevalent global barrier to publication and readership and thus providing wider audiences for research. Evidence suggests that CDSR-published articles have higher rates of women first and last authors than non-CDSR medical journals, ostensibly due to these improved access aspects (12, 13). In a cross-sectional study of 589 CDSR reviews across medicine between 2019-2020, women represented 55% of first authors and 40% of last authors (12). In oncology specifically, the opportunity to contribute to high-quality scholarship is critical for promoting the advancement of women in this field globally to improve cancer health disparities. If equitable authorship cannot be achieved with a platform with minimal barriers to entry, this speaks about the challenges of increasing equity across all oncology research for women.

Given the known gender disparities in leadership and scholarship in oncology, it is critical to evaluate the potential of this platform to improve the existing inequities in that field. The overall goal of this study was to retrospectively and longitudinally assess global rates of women as first, last, and corresponding authors in oncology publications in the CDSR over a twenty-year period.

## Methods

### Database

**Figure 1:**
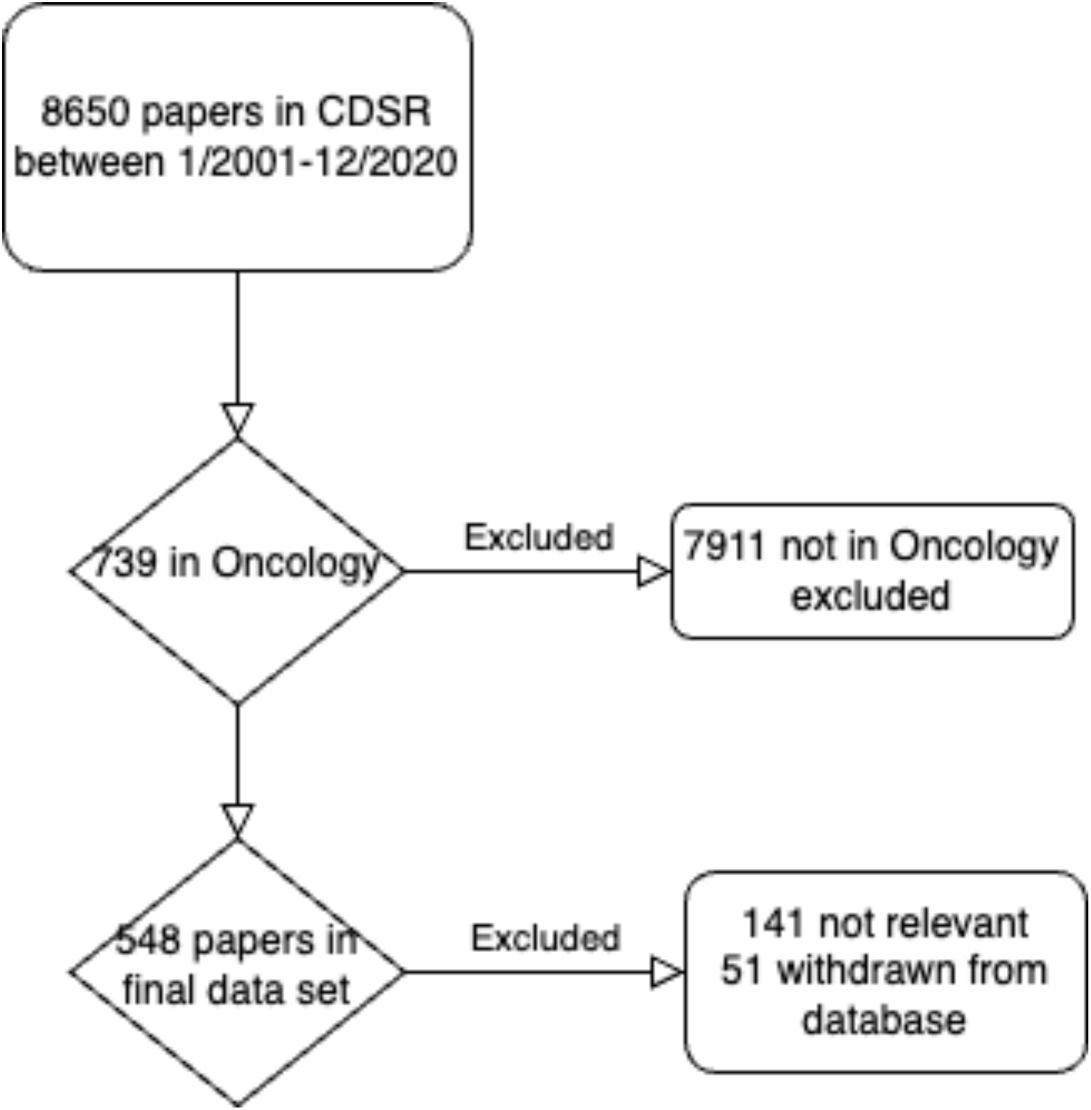
Overview of article selection for study inclusion.

The data in this study is publicly available; thus, IRB approval was not indicated. All CDSR records (n = 8650) published between January 1, 2001 to December 31, 2020 were reviewed (**Figure 1**) (11). Of these, 739 records (8.5%) reportedly dealing with oncology and hematology subjects were extracted (14). Following extraction, the subject relevance of each article was individually confirmed by two independent reviewers blinded to each other’s review and to study outcomes. All instances of discordance were refereed by the corresponding author.

### Analysis

A retrospective analysis was performed on the final database. Five coders manually extracted the following data for each article: first, last, and corresponding author name, gender, and country of work at time of publication; publication date, title, doi; and CDSR category. The first author was defined as the initial author in the authorship list. Last author was defined as the final author in the authorship list. The corresponding author was defined as the author whose contact information was listed on the cover page of the publication, regardless of position in the authorship list. All data analyzed in this study corresponds to the first, last, and corresponding authors, irrespective of the number of authors for each manuscript. The data for each included article was independently reviewed for accuracy by at least two study team members blinded to all data analyses. Instances of discordance were resolved by the corresponding author, who was blinded to the data analysis at time of decision.

Several methods were used to confirm the author’s gender. Gender was dichotomized as either “man” or “woman”. In the majority of cases, self-reported identity data for gender was not available. As follows, gender was defined by culturally driven assignment of names associated with gender on a binary spectrum (e.g., “Mary” as woman, “John” as man), as commonly applied in previously published authorship reviews (15). Names that traverse traditional gender assignments (i.e., “Kris”, “Bobby”) were verified by examining publicly available professional profiles (i.e., online academic profiles, professional social media profiles such as LinkedIn, etc.). Where available, preferred pronouns in these profiles were applied to assign gender. For authors listed with initial(s), other publications by the author(s) available on internet search engines (e.g., NCBI Pubmed) were independently evaluated to determine gender assignment. Gender was verified using Gender Checker and genderize.io, these tools utilize artificial intelligence to predict a person’s gender based on name (16, 17). Discrepancies arising in gender assignments between different blinded reviewers were resolved by discussion with a third blinded reviewer from the research team. All blinded reviewers are listed authors in this manuscript. The corresponding author was blinded to all aspects of gender assignment.

### Patient and public involvement

There was no involvement of patients or the public in this study as it was a retrospective review of a database.

### Statistical analysis

Statistical analyses were conducted using R version 4.0.4 (R Foundation for Statistical Computing, Vienna, Austria) (18). Author gender proportions were compared using one-sample binomial tests of proportions (19). To minimize bias in the authorship distribution by country, countries that had <10 authors in any role (2% of the dataset) were excluded. The one sample z-test of proportions was applied to compare individual author country assignment and author gender distribution. Changes in the percentage of women authors over time from 2001-2020 were analyzed using the Cochran-Armitage trend test (19). In all analyses, *p* < 0.05 was considered statistically significant.

## Results

### Dataset

One hundred forty-one articles were excluded from the study due to unanimous consensus on subject matter relevance. Fifty-one articles withdrawn from the CDSR due to quality concerns, (such as conclusions no longer relevant or up-to-date) were additionally excluded. The final data set consisted of 548 records.

### Overall authorship distributions

The overall gender distribution of authorship positions is summarized in **Table 1**. Women were first authors for 52.6% of publications (n=275), while men were first authors for 47.4% (n=248). Among corresponding authors, 50.9% (n=269) were women, and 49.1% (n=260) were men. Women represented 39.3% (n=207) of last authors, significantly lower than that of men at 60.7% (n=320) (*p <* 0.001).

**Table 1:** Cumulative gender distribution by authorship position.

Authorship	Women n(%)	Men n(%)	*p* value ^a^
**First**	275 (52.6)	248 (47.4)	0.16
**Corresponding**	269 (50.9)	260 (49.1)	0.67
**Last**	207 (39.3)	320 (60.7)	<0.001^*^

*p<0.05 is considered significant.

^a^Analysis performed via two-tailed z test of proportions.

### Editorial groups

The final database included reviews from eight distinct editorial groups, including Breast Cancer (n=62), Childhood Cancer (n=33), Colorectal Cancer (n=92), Gynecologic Cancer (n=98), Hematological Malignancies (n=51), Hematology (n=13), Lung Cancer (n=26), Neuro-oncology (n=26), Orphan Cancer (n=38) (cancer topics that do not fit into the other editorial groups), and Palliative and Supportive Care (n=97). The full breakdown of female authorship by editorial group and authorship position appears in **Table 2**.

**Table 2:** Author position by gender across CDSR editorial groups.

Authorship Position	First			Corresponding			Last		
	Women n (%)	Men n (%)	*p*	Women n (%)	Men n (%)	*p*	Women n (%)	Men n (%)	*p*
**Breast Cancer**	36 (58.1)	26 (41.9)	0.11	35 (56.5)	27 (43.6)	0.21	26 (41.9)	36 (58.1)	0.11
**Childhood Cancer**	19 (57.6)	14 (42.4)	0.32	16 (48.5)	17 (51.5)	1.0000	19 (57.6)	14 (42.4)	0.32
**Colorectal Cancer**	28 (30.8)	63 (69.2)	<0.001^*^	23 (25.0)	69 (75.0)	<0.001*	21 (23.6)	68 (76.4)	<0.001^*^
**Hematological Malignancies**	25 (50.0)	25 (50.0)	1.00	31 (60.8)	20 (39.2)	0.05	19 (37.3)	32 (62.8)	0.02^*^
**Hematology**	6 (46.2)	7 (53.9)	1.00	9 (69.2)	4 (30.8)	0.12	8 (61.5)	5 (38.5)	0.43
**Lung Cancer**	15 (57.7)	11 (42.3)	0.41	15 (57.7)	11 (42.3)	0.41	14 (53.9)	12 (46.2)	0.78
**Gynecologic Cancer**	63 (67.7)	30 (32.3)	<0.001^*^	62 (63.3)	36 (36.7)	<0.001^*^	49 (51.0)	47 (49.0)	0.89
**Neuro-oncology**	9 (34.6)	17 (65.4)	0.05	10 (38.5)	16 (61.5)	0.17	5 (19.2)	21 (80.8)	<0.001^*^
**Orphan Cancer**	21 (55.3)	17 (44.7)	0.49	19 (50.0)	19 (50.0)	1.0	12 (31.6)	26 (68.4)	0.003^*^
**Pain, Palliative and Supportive Care**	55 (56.1)	43 (43.9)	0.12	52 (53.6)	45 (46.4)	0.39	37 (37.4)	62 (62.6)	<0.001^*^

*p<0.05 is considered significant.

### Authorship country of origin

Distribution of female authorship by country of origin is presented in **Table 3**. Countries with fewer than 10 total records (Bahrain, Belgium, Colombia, Denmark, Finland, France, Greece, Ireland, Israel, Iceland, Malawi, Singapore, Spain, Switzerland, and Turkey) are not shown. Countries with the highest percentages of female first authors included the Netherlands (68.8%), UK (59.5%), Australia (56.5%), and Canada (52.6%). Italy (4.5%) and China (13.0%) had the lowest percentages of women first authors, though total number of records in the latter cases was low. The Netherlands (63.0%), United Kingdom (58.9%), Brazil (57.1%), and Australia (52.5%) had the highest percentages of female corresponding authors. Italy (0%), China (17.9%), and USA (34.6%) had the lowest percentages of female corresponding authors. Brazil (75.0%), the Netherlands (56.5%), and Australia (50.8%) had the highest percentages of women in last author positions. The lowest percentages of female last authors were seen from Italy (0%) and China (13.0%).

**Table 3:** Authorship gender distribution by country.

	First			Corresponding			Last		
	Women n (%)	Men n (%)	*p*	Women n (%)	Men n (%)	*p*	Women n (%)	Men n (%)	*p*
**Australia**	35 (56.5)	27 (43.5)	0.21	32 (52.5)	29 (47.5)	0.72	30 (50.8)	29 (49.2)	1.00
**Brazil**	4 (40.0)	6 (60.0)	0.65	8 (57.1)	6 (42.9)	0.71	9 (75.0)	3 (25.0)	0.04*
**Canada**	10 (52.6)	9 (47.4)	1.00	8 (40.0)	12 (60.0)	0.34	8 (44.4)	10 (55.6)	0.74
**China**	3 (13.0)	20 (87.0)	<0.001*	5 (17.9)	23 (82.1)	<0.001*	3 (13.0)	20 (87.0)	<0.001*
**Germany**	25 (43.1)	33 (56.9)	0.19	28 (49.1)	29 (50.9)	1.00	24 (47.1)	27 (52.9)	0.69
**Italy**	1 (4.5)	21 (95.5)	<0.001*	0 (0)	22 (100)	<0.001*	0 (0)	17 (100)	<0.001*
**UK**	150 (59.5)	102 (40.5)	<0.001*	149 (58.9)	104 (41.1)	<0.001*	84 (34.0)	163 (66.0)	<0.001*
**USA**	10 (37.0)	17 (63.0)	0.10	9 (34.6)	17 (65.4)	0.05	9 (34.6)	17 (65.4)	0.05
**Netherlands**	33 (68.8)	15 (31.3)	<0.001*	29 (63.0)	17 (37.0)	0.02*	26 (56.5)	20 (43.5)	0.29

*p<0.05 is considered significant.

Both China and Italy demonstrated a consistent and statistically significant discrepancy between female and male first, corresponding, and last authorship (*p* < 0.001 in all cases). Also, despite highest rates of female authors amongst country comparisons, the Netherlands and UK demonstrated a statistically significant disparity favoring male first authors (*p* < 0.001 in both cases) and male corresponding authors (*p* = 0.02 in UK; *p* < 0.001 in the Netherlands), and the UK continued this trend with disparity strongly favoring male last authors (*p* < 0.001). Notably, the USA trended toward significant disparity favoring male first authors (*p* = 0.10), corresponding authors (*p* = 0.05), and male last authors (*p* = 0.05).

### Authorship distribution over time

**Figure 2:**
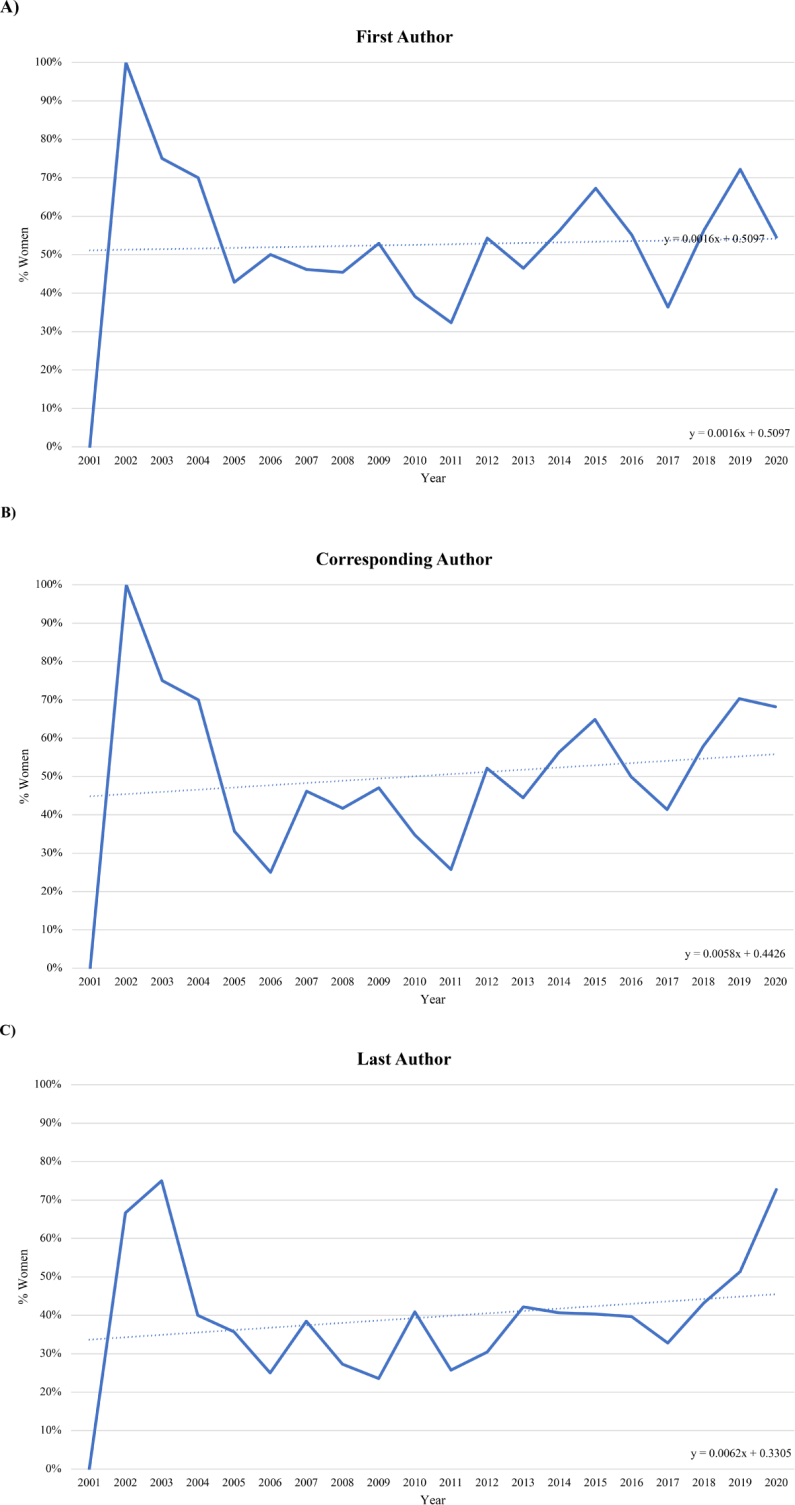
Trends in authorship gender from 2001-2020. Gender distribution of first, corresponding, and last authors by the Cochrane-Armitage trend test, dotted lines represent linear trend lines. Years with less than 5 manuscripts included were 2 papers in 2001, 3 papers in 2002, and 4 papers in 2003. **(A)** Women as first authors (y=0.061x+0.5097, *p* = 0.20). **(B)** Women as corresponding authors (y=0.0058x+0.4426, *p* = 0.02). **(C)** Women as last authors (y=0.0062x+0.3305, *p* = 0.04).

Though there was a trend toward increase in overall percentage of women authors during the period 2001-2020, this was not statistically significant (**Figure 2**). When assessed by authorship position, we found that the distribution of female corresponding authors (*p* = 0.04) and last authors (*p* = 0.02) significantly increased between 2001 and 2020. However, the distribution of female first authors did not change significantly across the study period (*p =* 0.25).

## Discussion

These results highlight measurable patterns of authorship gender disparity for hematology/oncology articles in the CDSR from 2001-2020. While the overall percentage of women first authors (52.6%) and corresponding authors (50.9%) did not differ significantly from that of men, the proportion of women last authors was significantly lower (39.3%), and this disparity was highly statistically significant. Notably, the first and last author positions, independent of corresponding authorship, are frequently assumed to represent the primary driver and thought leader, respectively, of a publication. Citations more frequently reference first and last authors, contributing to name recognition and global academic reputation/stature. Thus these results suggest authorship disparities that could have significant bearing on academic advancement of women in hematology/oncology and merits further prospective study.

We also found that the proportion of women as corresponding and last authors increased significantly during our study period. These trends are an encouraging indicator that female representation in oncology research and in leadership roles is increasing, yet more work is still needed to achieve gender equity in the field.

A study looking at 5302 authors in 608 articles published in *JCO Global Oncology* from October 2015- March 2020 found that women accounted for 41.4% of first authors, and 32.1% of last authors (percentages lower than we found in the CDSR) (3). This may relate to the fact that this was a single-journal study and that the global South and developing nations were more represented the *JCO Global* dataset used than in the CDSR. However, our trends within the global North strongly recapitulate the trends for reduced representation of women in key academic authorship positions and extend the findings to developed nations where economics alone is inadequate to explain the disparity.

Our results highlight that authorship distributions by gender differ dramatically by country of origin, suggesting other key societal and perhaps cultural variables at play. Countries such as the Netherlands and Australia consistently showed equitable representation in first, corresponding, and last authorship, with the proportion of female authors being above 50% in every authorship position. Other countries such as Italy and China had uniformly low rates of female authorship, with all percentages being less than 20%. Also, some countries such as China and Italy showed statistically significant and pervasive disparities in women in all authorship positions, a finding spanning across the perceived development status of China and Europe and suggesting other variables at play. The study by Hornstein et al. in *JCO Global* found that women were more likely to be the first authors in high-income countries (HIC, as defined by World Bank criteria of 2020), and less likely to be last authors in lower-middle-income (LMIC) and low-income countries (LIC) (3). While our database represents largely HIC and non-LMIC/LIC countries, the finding that women are represented at lower rates as first and corresponding authors is consistent. Notably, in our study reviewing 1581 authors through the CDSR database over a 20-year period, the USA and other countries traditionally perceived as “developed” showed significant disparity trends which paralleled those found in other studies in the “developing” world (3). More studies are needed to elucidate the reasons for gender gaps in scholarship by nation, and explore effective interventions in the nations with the starkest disparities.

While few studies publish authorship trends by country, our findings are consistent with previously published trends in gender inequities by academic rank. For example, in Italy, which falls in the category of HIC, women represent 40.9% of assistant professors, 25.0% of associate professors, and 13.3% of full professors in academic medicine (20).

Addressing global inequities in academic representation is critical to promoting high-impact scholarship for women in oncology. A bibliometric analysis of major oncology journals by Dalal et al. found that, in the 50 most cited articles in each year between 1990 and 2017, women were less likely to be first (26.5%) or last (19.9%) authors (2). Given that citations typically refer to the first author either within the context of multiple authors cited or as the dominant in a group of authors (e.g. “Dalal et al) or to the last author (e.g. “the (senior author) group”), this lack of equity of representation of women in first and senior author positions directly adversely affects citation impact and recognition of the woman author’s contributions in a dominant role in these manuscripts. This ultimately impacts women’s potential for promotion by minimizing their recognized scholarly contributions in tenure decisions (9). Another study found that female researchers favor publication in open-access journals compared to their male counterparts who pursue publication in non-open access journals, some of which have significantly higher impact factors than open-access journals (21). These trends may further exacerbate the academic promotion gap as institutions move toward quantifiable metrics which include point values assigned for publications in higher impact journals, higher *h*-indexes, etc. Substantive efforts are necessary to improve the publication disparities highlighted in this study and achieve gender parity in oncology. Of note, these issues perpetuate themselves across many areas of academic medical publications as demonstrated by a similar study demonstrating gender inequity in publications in exercise and rehabilitation (22).

With time, increasing attention has been directed toward improving female representation in medicine, though additional initiatives are necessary to target authorship in particular. The first step toward addressing these gender gaps should be identifying and quantifying disparities via larger-scale studies like ours and through department- and institution-level data. This local data on authorship and funding can be stratified by gender to highlight existing gaps at one’s own academic center and publicize such disparities (23). Notably, many male physicians and researchers remain unaware of the gender inequities that persist in medicine, which may contribute to decreased willingness to support gender equity initiatives (24). Thus, promoting awareness and educating male physicians with data on relevant authorship disparities is essential to develop male allyship for gender equity efforts in academia. Such data is also valuable for identifying areas for intervention at individual, team and departmental levels. Effective initiatives may include developing systems to support authorship collaboration, encouraging shared first and last authorship, and providing mentorship throughout the writing and publication process (Allen et al., Silver et al) (23, 25). The efficacy of reducing publication costs for female and minority authors could also be explored, particularly for journals with greater gender disparities.

Broader efforts to promote inclusion of women in academic medicine may also improve female scholarship via reduction of implicit biases in review and publication processes. Therefore, it remains important to scale-up existing strategies such as mandatory unconscious bias training for leaders (26), setting term limits for leadership roles (27), and minimizing requests for involvement in low-yield institutional service (e.g., unrecognized committee roles with no opportunities for professional advancement) that detract from research productivity (28). Additional research is warranted to quantify the effectiveness of these efforts.

This study’s strengths include the longitudinal nature of data over 20 years. Additional strengths include the global representation of articles by country rather than conglomerate representation by WHO income level classification. This has allowed an evaluation of disparity trends that suggest factors outside of simple economic status (e.g. USA trends, disparities data for Italy and China being similar despite vastly differing economic status).

Limitations of this study include that all articles derive from the CDSR and are, therefore, subject to the selection biases inherent to the database. Many regions of the world are not included or poorly represented in our dataset based on low rates of inclusion in CDSR, including many LMIC, resulting in small sample sizes which reduced our power to draw conclusions in these areas. Rigorous research into global oncology authorship trends by country are warranted, as gender norms likely vary by culture and country. Given the lack of access to self-reported genders in a retrospective database study, our study utilized name as a proxy for gender identity. This proxy approach is limited due to variable cultural norms, variations in public author profile availability by region, and misclassification for authors who may identify as gender non-conforming or non-binary. Additionally, the generize.io and gender checker tools have varying accuracy depending on the name, which introduces the possibility of error. As institutional websites often do not include pronouns, we cannot report the percentage of authors in this study identifying as gender non-conforming or non-binary. The assumption that last author is perceived as the leader of the study for academic advancement may also not be valid across countries or cultural norms. Additionally, due to the absence of self-reported data on race of the authors included in the analysis, this study cannot address race and racism as factors impacting opportunities for equitable authorship in oncology. Finally, the high number of analyses included increases the potential for type I error.

Overall, this study illuminates key patterns of gender inequity in oncology authorship spanning over 2 decades in a pattern that transcends economic variables and geographic region but is highly consistent across authorship categories by country of origin. Our data further suggests that, while there has been significant improvement in gender inequity in key authorship roles in the last two decades, some countries continue to demonstrate pervasive disparities in female authorship across all categories. Gender imbalance in academic authorship reduces the diversity of intellectual contribution to advancement of the field, risks skewing research priorities, contributes to the loss of academic talent, and may perpetuate discriminatory practices including pervasive unconscious biases that limit women from ascending to leadership roles in academic oncology. Systematic, substantive changes focused on early and consistent correction of the key variables influencing first and senior authorship on research publications is likely to be a key contributor to enhanced global gender equity in academic hematology/oncology.

## Data Availability

The raw data supporting the conclusions of this article will be made available by the authors, without undue reservation.
